# Long-Lasting and Additive Analgesic Effects of Combined Treatment of Bee Venom Acupuncture and Venlafaxine on Paclitaxel-Induced Allodynia in Mice

**DOI:** 10.3390/toxins12100620

**Published:** 2020-09-28

**Authors:** Daxian Li, Ju Hyuk Yoo, Sun Kwang Kim

**Affiliations:** 1 Department of Science in Korean Medicine, Graduate School, Kyung Hee University, Seoul 02447, Korea; lidaxian721@naver.com; 2 Department of East-West Medicine, Graduate School, Kyung Hee University, Seoul 02447, Korea; impera27@naver.com; 3 Department of Physiology, College of Korean Medicine, Kyung Hee University, Seoul 02447, Korea

**Keywords:** bee venom acupuncture, venlafaxine, combination therapy, paclitaxel, allodynia

## Abstract

Paclitaxel, a primary chemotherapeutic agent used to treat numerous solid malignancies, is commonly associated with debilitating peripheral neuropathy. However, a satisfactory gold-standard monotherapy for this neuropathic pain is not currently available. A combination strategy of two or more medications with different properties may achieve more beneficial effects than monotherapy. Thus, we investigated the analgesic efficacies and spinal mechanisms of the combination strategy, including bee venom acupuncture (BVA) and venlafaxine (VLX) against paclitaxel-induced allodynia in mice. Four intraperitoneal infusions of paclitaxel on alternating days (2 mg/kg/day) induced cold and mechanical allodynia for at least 1 week as assessed using acetone and the von Frey hair test, respectively. Co-treatment of BVA (1.0 mg/kg, s.c., ST36) with VLX (40 mg/kg, i.p.) at the medium dose produced a longer-lasting and additive effect than each monotherapy at the highest dose (BVA, 2.5 mg/kg; VLX, 60 mg/kg). Spinal pre-administration of idazoxan (α_2_-adrenergic receptor antagonist, 10 μg), methysergide (mixed 5-HT_1_/5-HT_2_ receptor antagonist, 10 μg), or MDL-72222 (5-HT_3_ receptor antagonist, 10 μg) abolished this analgesia. These results suggest that the combination therapy with BVA and VLX produces long-lasting and additive analgesic effects on paclitaxel-induced allodynia, via the spinal noradrenergic and serotonergic mechanism, providing a promising clinical strategy.

## 1. Introduction

Paclitaxel, a derivative of *Taxus brevifolia*, is recommended worldwide as one of the standard antineoplastic medications against various types of carcinomas, including breast, lung, and ovarian cancers [1]. Nevertheless, 7 out of 10 patients receiving paclitaxel develop a devastating dose-limiting side effect, paclitaxel-induced peripheral neuropathy [2]. The representative clinical manifestations of this unwanted neuropathy include overt numbness, tingling, ongoing pain, and allodynia, most of which progress symmetrically from the feet and hands [3,4]. This burdensome neurotoxic state can be sufficiently distressing to lead to temporary cessation or even termination of life-saving therapy, thereby ultimately affecting the survival likelihood [1,4]. Unfortunately, a satisfactory gold-standard monotherapy protocol is not currently available [5]. Hence, there is an urgent need to identify favorable co-administration strategies for two or more medications that are already used in the clinic, which may offer considerable beneficial analgesia without unexpected adverse effects [6,7].

Bee venom acupuncture (BVA), also referred to as apitherapy, is a well-known traditional treatment approach that involves stimulation of acupoints with diluted bee venom, and has been used to exert healing effects in a variety of painful conditions in Korean medicine, such as lower back pain and post-stroke pain [8,9]. Recent clinical pilot practices revealed that BVA can reduce chemotherapy-induced pain levels, and then increase patients’ quality of life (QOL) [10,11]. In addition, we and others previously elucidated that BVA at ST36 (Zusanli) acupoint could significantly mitigate the neurotoxic symptoms of chemotherapy-treated rodents, primarily through spinal α-adrenergic receptor (α-AR)-mediated mechanisms [12,13,14]. The ST36 acupoint is located on the tibialis anterior muscle of the leg, and its functions in traditional oriental medicine are to fortify the spleen and stomach, replenish qi and nourish blood, clear and activate meridians, and nourish the channels [15]. Lots of pre-clinical works indicated that stimulations applied on this acupoint (e.g., BVA, electro-acupuncture, laser-acupuncture, or moxibustion) can produce significant pain-relieving effects under diverse conditions, such as neuropathic, incision, or inflammatory pain state [16,17,18,19].

Venlafaxine (VLX) is a representative serotonin (5-HT) and noradrenaline reuptake inhibitor (SNRI), and is increasingly used as a first-line treatment against diverse chronic neuropathic pain pathologies [1,20,21]. In fact, VLX shows analgesic properties mainly by increasing the amounts of noradrenaline and serotonin at the spinal cord level [22]. In our recent study, a single intraperitoneal treatment with VLX (40 mg/kg; i.p.) markedly ameliorated cold and mechanical allodynia elicited by another anticancer agent, oxaliplatin [23]. However, the analgesic effect of VLX on paclitaxel-evoked neuropathic pain in rodents has not been determined yet, and few studies have revealed the precise analgesic mechanism underlying this antidepressant.

In this context, we first verified whether BVA or VLX monotherapy has a dose-dependent analgesic efficacy on paclitaxel-induced cold and mechanical allodynia in mice. Second, we explored whether the combination strategy containing both intermediate dosages of BVA and VLX could have more beneficial analgesic effects than that of each monotherapy. Finally, by performing direct intrathecal administration of antagonists, we sought to clarify which spinal adrenergic and/or serotonergic receptors mediate the combination therapy analgesia.

## 2. Results

### 2.1. Cold and Mechanical Allodynia Following Repeated Paclitaxel Treatments

**Figure 1:**
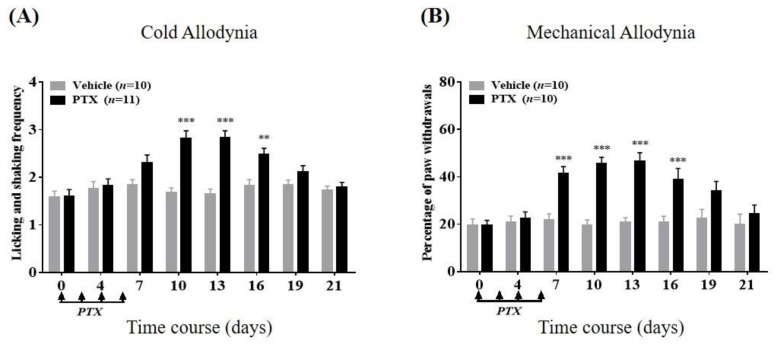
Change over time in repeated paclitaxel (PTX) treatment-induced cold (**A**) and mechanical (**B**) allodynia in mice. On days 0, 2, 4, and 6 (4 arrows), PTX or vehicle was administered (i.p.). Mice were subjected to measurement of allodynia just before and between day 4 to day 21 after the first dose of PTX (on the timeline: days 0, 4, 7, 10, 13, 16, 19, and 21, respectively). In the acetone test, the number of licking and shaking motions in 30 s were counted. In the von Frey assay, the percentage was used to express the result: (number of tests accompanied by withdrawals) × 100/(number of total tests). Data are expressed as mean ± S.E.M.; ** *p* < 0.01, *** *p* < 0.001, vs. vehicle by Bonferroni post-test after two-way analysis of variance (ANOVA).

Four intraperitoneal infusions of paclitaxel on alternating days (2 mg/kg/day; days 0, 2, 4, and 6) significantly exaggerated the licking and shaking frequency of the hind paw in response to 10 μL acetone spray (Figure 1A), indicating the development of cold allodynia. In addition, the same paclitaxel interventions significantly escalated the withdrawal percentage of the hind paw in response to the von Frey filament with 0.4 g bending force (Figure 1B), signifying a symptom of mechanical allodynia. As seen in Figure 1, significant cold allodynia was observed from day 10 to day 16 (*p* < 0.01, day 16; *p* < 0.001, day 10 and 13, respectively), and significant mechanical allodynia was observed from 7 to 16 days after the initiation of paclitaxel treatment (*p* < 0.001, days 7, 10, 13, and 16, respectively). Accordingly, for the next experiments, the pain-relieving effects of both monotherapy and combination therapy were tested during days 10–16 when both cutaneous allodynic symptoms were markedly exhibited.

### 2.2. Relieving Effects of ST36 Treatment with BVA on Paclitaxel-Induced Cold and Mechanical Hypersensitivity

**Figure 2:**
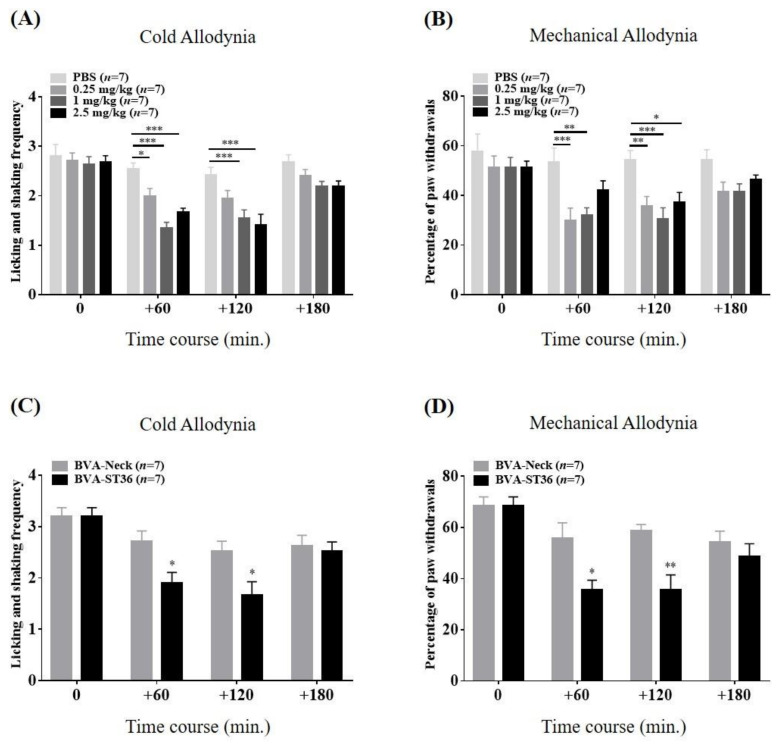
Time course of the relieving effects of ST36 treatment with bee venom acupuncture (BVA) on paclitaxel-induced cold (**A**) and mechanical (**B**) allodynia in mice. Mice were divided arbitrarily into four groups. “0.25,” “1.0,” and “2.5 mg/kg” referred to the doses of BV injected at ST36. (**C**,**D**) illustrated the analgesic effects of 1.0 mg/kg of BVA at ST36 (BVA-ST36) versus at the neck, a different body area (BVA-Neck), on cold and mechanical allodynia. Behavioral sensitivity was assessed four times; just prior to the injection of BV and resumed at 60-min intervals for 3 h after treatment (on the timeline: 0, 60, 120, and 180, respectively). Data are expressed as mean ± S.E.M.; * *p* < 0.05, ** *p* < 0.01, *** *p* < 0.001, vs. control (PBS; **A** and **B**); * *p* < 0.05, ** *p* < 0.01, vs. BVA-Neck group (**C** and **D**) by Bonferroni post-test after two-way ANOVA.

To verify the anti-allodynic effects of BVA on paclitaxel-induced peripheral neuropathy, three different doses of BV or vehicle (PBS) were injected subcutaneously at ST36 of mice showing symptoms of hypersensitivity (days 10–16; 0.25, 1.0, and 2.5 mg/kg; s.c.). In terms of cold allodynia, 1.0 and 2.5 mg/kg BV ameliorated the acetone-elicited withdrawal frequency, lasting for 120 min (Figure 2A). The lowest dose BV (0.25 mg/kg) alleviated cold hypersensitivity only at the 60 min time point. In the von Frey test, compared to the PBS-treated mice, the low and medium BVA treatment groups (0.25 and 1.0 mg/kg) showed a markedly decreased withdrawal percentage of the hind paw at 60 min and 120 min (0.25 mg/kg: 60 min, *p* < 0.001; 120 min, *p* < 0.01 and 1.0 mg/kg: 60 min, *p* < 0.01; 120 min, *p* < 0.001. Figure 2B). However, the highest dose group (2.5 mg/kg) showed a similar effect only at the 120 min time point. These results clearly demonstrate that BVA treatment ameliorates cold and mechanical allodynia generated by paclitaxel, for which the optimum dosage is 1.0 mg/kg. Next, Figure 2C,D evaluated the effect of 1.0 mg/kg of BVA at ST36 (BVA-ST36) versus the neck, a different body area (BVA-Neck), on cold and mechanical measurements. At the 60 and 120 min points, significant differences were observed between the BVA-ST36 and BVA-Neck groups in both behavioral assessments. Thus, mice dosed with BV directly on the ST36 acupoint showed a more favorable analgesic effect than that produced by neck area administration on paclitaxel-induced allodynia.

### 2.3. Relieving Effects of VLX on Paclitaxel-Induced Cold and Mechanical Hypersensitivity

**Figure 3:**
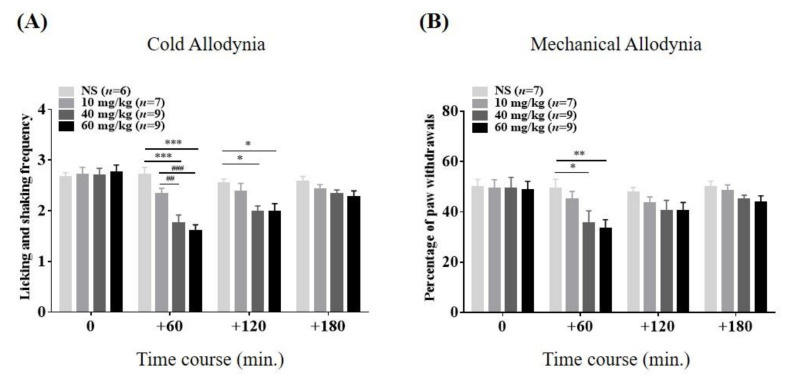
Time course of the relieving effects of venlafaxine (VLX) on paclitaxel-induced cold (**A**) and mechanical (**B**) allodynia in mice. Mice were assigned arbitrarily into four groups. “10,” “40,” and “60 mg/kg” refer to the doses of VLX treatment. Both measurements were performed four times; just before the injection of VLX and at 60-min intervals for 3 h after treatment (on the timeline: 0, 60, 120, and 180, respectively). Data are expressed as mean ± S.E.M.; * *p* < 0.05, ** *p* < 0.01, *** *p* < 0.001, vs. control (NS); ## *p* < 0.01, ### *p* < 0.001, vs. 10 mg/kg VLX group by Bonferroni post-hoc test after two-way ANOVA.

In a subsequent experiment, we evaluated the relieving actions of VLX on cold and mechanical allodynia induced by repeated administration of paclitaxel. After being assigned arbitrarily into four subgroups, mice with neuropathy were treated with VLX (10, 40, and 60 mg/kg, i.p.) or vehicle (NS). VLX at intermediate and highest doses (40 and 60 mg/kg) were shown to be effective in cold and mechanical allodynia, lasting for 120 and 60 min, respectively (Figure 3A,B). In terms of mechanical allodynia, the highest dose of VLX was shown to have a somewhat stronger action than the medium dose (NS vs. 60 mg/kg, *p* < 0.01 and vs. 40 mg/kg, *p* < 0.05). However, at the endpoint of the test, all VLX-treated groups showed negligible analgesic effects on both cold and mechanical hypersensitivity (180 min, *p* > 0.05, respectively). Hence, our results reveal that a single i.p. treatment of VLX produced anti-allodynic effects in a dose-dependent manner in this model.

### 2.4. Effects of Combination Therapy on Paclitaxel-Induced Cold and Mechanical Hypersensitivity

**Figure 4:**
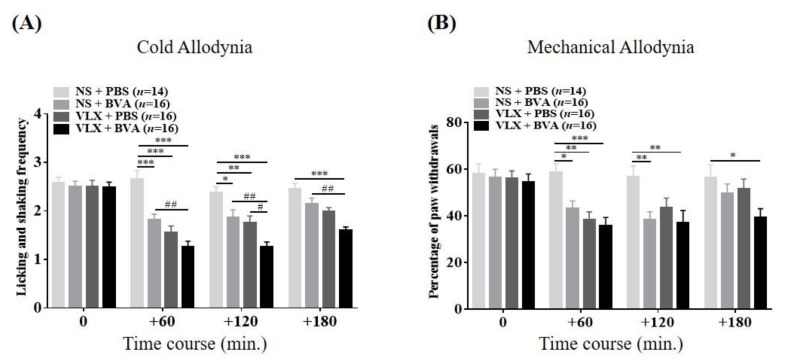
Elapsed time of the combined effects of BVA and VLX on paclitaxel-induced cold (**A**) and mechanical (**B**) allodynia. Mice were randomly assigned into four groups: I: NS + PBS; II: NS + BVA; III: VLX + PBS; IV: VLX + BVA. “PBS” and “NS” refer to the vehicle of bee venom (BV) or VLX, respectively. The combination therapy (VLX + BVA) included intermediate doses of both BV and VLX (BV, 1.0 mg/kg, s.c.; VLX, 40 mg/kg, i.p). Data are expressed as mean ± S.E.M.; * *p* < 0.05, ** *p* < 0.01, *** *p* < 0.001, vs. control (NS + PBS); # *p* < 0.05, ## *p* < 0.01, vs. combination group by Bonferroni post-hoc test after two-way ANOVA.

In a separate cohort of mice, the combined effects of a medium dose of BVA and VLX on cold and mechanical allodynia elicited by paclitaxel were examined. In the combination strategy group (VLX + BVA), mice in allodynic states underwent BVA (1 mg/kg, s.c.) and VLX (40 mg/kg, i.p.) administration simultaneously. As expected, in each assessment (Figure 4A,B), compared to the vehicle-treated (NS + PBS) mice, the analgesic actions of the monotherapy group (e.g., NS + BVA and VLX + PBS) lasted only for 120 min. On the other hand, the co-treatment strategy significantly reduced licking and shaking frequencies (cold allodynia) and the percentage of hind paw withdrawal (mechanical allodynia) until the 180 min point. It was also apparent that combination therapy showed markedly greater inhibitory effects than both monotherapy groups at the 120 min point in the acetone test. Thus, our data indicate that co-administration of BVA at 1.0 mg/kg and VLX at 40 mg/kg fully mitigates the paclitaxel-elicited cold and mechanical hypersensitivity, suggesting longer-lasting and additive effects over other monotherapy groups.

### 2.5. Spinal Mechanisms of Combination Therapy against Cold and Mechanical Allodynia

**Figure 5:**
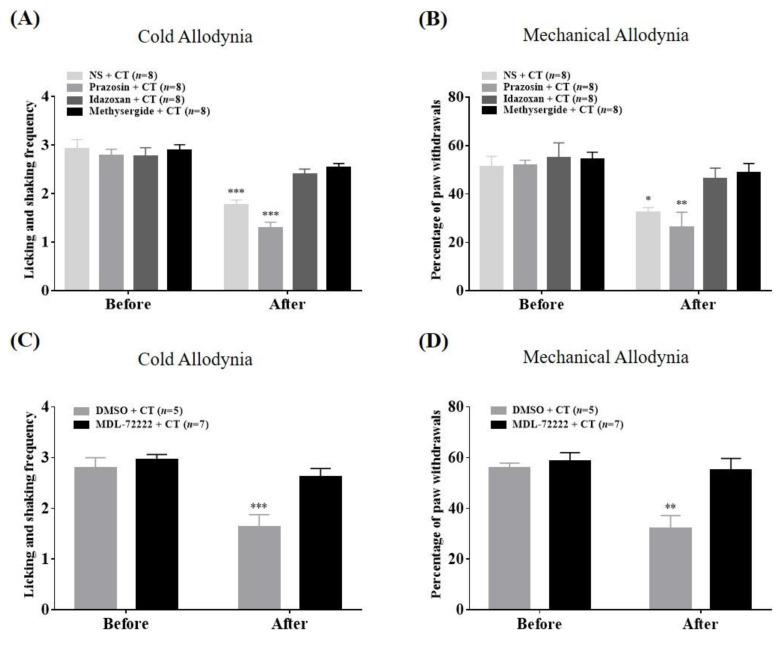
Effects of spinal adrenergic (**A**) and (**B**) and 5-HT (**C**) and (**D**) receptor antagonism on combination treatment analgesia in cold and mechanical allodynia. Mice were divided into six groups that received each antagonist or appropriate vehicle intrathecally (i.t.); normal saline (NS), 20% dimethyl sulfoxide (DMSO), prazosin, idazoxan, methysergide or MDL-72222 at 20 min before combination treatment (BVA, s.c., 1 mg/kg and VLX, i.p., 40 mg/kg). Acetone test and von Frey test were performed twice; just prior to the intrathecal injection (Before) and 60 min after combination therapy (After). Data are expressed as mean ± S.E.M.; * *p* < 0.05, ** *p* < 0.01, *** *p* < 0.001, vs. Before; by Bonferroni post-hoc test after two-way ANOVA.

To examine the analgesic mechanism of combination therapy against cold (Figure 5A,C) and mechanical (Figure 5B,D) allodynia at the spinal level, four types of α-adrenergic and 5-HT receptor antagonists were administered intrathecally (i.t.) 20 min before combination treatment (BVA, 1 mg/kg, s.c.; VLX, 40 mg/kg, i.p.). Pretreatment with each appropriate vehicle (NS and 20% DMSO, respectively) or α_1_-adrenergic receptor antagonist, prazosin, did not alter the analgesia from combination treatment (*p* > 0.05). However, mice pre-administered other antagonists (idazoxan, methysergide, MDL-72222) demonstrated no anti-allodynic effect of combination therapy. In other words, spinal α_2_-AR, 5-HT_1_, 5-HT_2_, and 5-HT_3_ receptors, but not the α_1_-AR subtype, mediate the analgesic effects of co-administered BVA and VLX on cold and mechanical allodynia generated by paclitaxel.

## 3. Discussion

Paclitaxel, a primary treatment for a variety of solid malignancies (e.g., lung, cervical, and ovarian carcinomas), is commonly associated with debilitating paresthesia, modest tingling, and severe persistent pain, which could necessitate dose reduction or termination of chemotherapy [3,24]. To date, despite numerous studies to identify ideal analgesics, no medication without any shortcomings (i.e., limited efficacy or adverse effects) has been introduced to the clinic [5,7]. One reason for this is that chemotherapy-induced peripheral neuropathy (CIPN) has diverse pathophysiological mechanisms [25]. As such, drug combination therapy involving two or more mechanisms may increase the possibility of achieving more promising pain alleviation [16,26].

BVA is a traditional pharmacopuncture method in Korea, and its pain relief efficacy is generally utilized for an array of musculoskeletal diseases (e.g., shingles, gout, and arthritis) [9,27,28]. A clinical case report has shown that a one-week treatment of BVA can improve the pain level as assessed by both the World Health Organization (WHO) CIPN grade and visual analog scale (VAS) [10]. Recently, we discovered that a single subcutaneous injection of BV or its main components (e.g., melittin or bvPLA2) at ST36 clearly mitigates allodynic symptoms generated by paclitaxel or oxaliplatin, which are mainly associated with the spinal noradrenergic mechanisms [12,13]. Meanwhile, VLX, which is an SNRI antidepressant, is recommended as a first-line medication for neuropathic pain in recent clinical guidelines [20,29]. Some clinical trials have concentrated on its pain-relieving effects in CIPN patients, and have elucidated that VLX (75 mg/day; p.o.) has beneficial effects against taxane- or platinum-based CIPN [30,31]. It is clear that spinal adrenergic and serotonergic pathways participate in VLX analgesia in neuropathic pain and inflammatory pain [32,33,34]. Consequently, we hypothesized that the combination of BVA with VLX, which belong to different categories of treatments (apipuncture vs. antidepressant), delivered through different routes (s.c. vs. i.p.), involved in different analgesic mechanisms (adrenergic vs. serotonergic and/or adrenergic), might exert more vigorous or enduring effects against paclitaxel-induced CIPN in mice.

First, we verified the time- and dose-dependent effects of BVA and VLX monotherapy on paclitaxel-induced allodynia. In the mechanical assay, at 60 min after treatment, the highest dose of BV (2.5 mg/kg) showed an inferior effect to that of the lowest (0.1 mg/kg) and medium (1.0 mg/kg) doses. These results were in agreement with the outcomes of our previous trial, despite the difference in BVA-treated acupoints and behavioral tests (ST36 vs. GV3 and von Frey test vs. tail immersion test) [35]. Next, we found that the effects of both the medium and highest dose of VLX lasted until 120 min after treatment in the acetone test. However, in the von Frey test, the analgesic effects of both 40 mg/kg and 60 mg/kg groups became negligible at 120 min. Similarly, as elucidated from our own trial and others [23,36], a single treatment of VLX (s.c. or i.p.) alleviated the cold allodynia more prominently than the mechanical allodynia generated by oxaliplatin, suggesting that VLX-induced analgesia could be more sensitive to cold rather than mechanical pain sensation, at least in CIPN models.

Second, we focused on the potentially more favorable efficacy of a combination strategy containing an intermediate dose of BVA (1 mg/kg; s.c.) and VLX (40 mg/kg; i.p.). Combination treatment showed a more enduring analgesic effect up to the endpoint of measurement (180 min) (Figure 4) than that of the BVA or VLX monotherapy groups at any dose (Figure 2 and Figure 3). In addition, compared to the monotherapy groups, mice treated with the combination therapy demonstrated marked inhibitory effects at the 120-min point in the acetone assay. Of note, a number of clinical and pre-clinical trials suggested that adding two or more medications to the co-treatment strategy seemingly did not always achieve a more vigorous analgesia and instead caused unexpected adverse effects [6,37,38]. Taken together, our data indicate that co-administration of BVA and VLX may be a promising novel strategy, at least in paclitaxel-induced allodynia.

As mentioned above, our data suggest that BVA plus VLX induced analgesia via spinal α_2_-AR and 5-HT_1_/5-HT_2_ and 5-HT_3_ receptor mechanisms. In our recent assessment, BVA analgesia in the paclitaxel model was deeply modulated by spinal α_2_-AR [13]. Meanwhile, VLX also showed a relieving effect on diverse pain models via spinal 5-HT receptors and/or α_2_-AR activation [23,32,39]. Serotonin is one of the most essential neurotransmitters in the descending pain inhibitory system [40], and the 5-HT_1_, 5-HT_2_, and 5-HT_3_ receptor subtypes are densely distributed in the superficial dorsal horn [41]. Thus far, many studies have suggested that activation of these receptors produced considerable analgesia in a nerve injury model (5-HT_1_ receptor) [42], pyrazolinone-induced pain model (5-HT_2_ and 5-HT_3_ receptors) [43], and SART (specific alternation of rhythm in environmental temperature)-stress model (5-HT_3_ receptor) [44]. In addition, the interaction of the noradrenergic-serotonergic pathway in pain modulation has been suggested. For instance, the analgesic effects of the 5-HT agonists were reversed by systemic administration of noradrenergic neurotoxin, N-2-chloroethyl-N-ethyl-2-bromobenzylamine (DSP4), [15]. A recent study discovered that both adrenergic and serotonergic signaling modulated the diffuse noxious inhibitory controls (DNIC) in an osteoarthritis pain model [45]. Another study demonstrated that yohimbine (α_2_-AR antagonist) abolished the dose-dependent suppressive effect of serotonin on the activity of spinal wide-dynamic-range (WDR) neurons [46].

In the present research, we used neuropharmacological and behavioral approaches to demonstrate the additive and long-lasting analgesic effects of BVA and VLX co-treatment in paclitaxel-injected mice and its spinal noradrenergic (α_2_-AR) and serotonergic (5-HT_1_/5-HT_2_, and 5-HT_3_ receptors) mechanisms. Our previous studies suggest that VLX or BVA monotherapy’s action could last only for 60 min or 120 min, and was mediated by spinal α_2_ or 5-HT_3_ mechanisms [12,16,23]. Although we could not understand all the details, the present results let us speculate that 5-HT_1_ and 5-HT_2_ pathways may be involved in the long-lasting effect of the combination therapy. In future studies using in vivo extracellular recordings in the spinal cord, it would be of high interest to elucidate how such combined therapy modulates the nociceptive transmissions of spinal projection neurons, such as WDR neurons [47]. Another limitation of this study is the use of only male mice. Since the differences in pain perception and tolerance between the sexes have been demonstrated [48], further studies are needed to examine whether the combination therapy of BVA and VLX also induces the comparable analgesic effect in female rodent models [49].

## 4. Conclusions

In summary, our results demonstrated that combination therapy containing 1.0 mg/kg of BVA and 40 mg/kg of VLX can relieve the cold and mechanical allodynia generated by multiple injections of paclitaxel in mice. This combination strategy provided longer-lasting and additive analgesic action than each monotherapy (BVA or VLX) at any dose, via spinal α_2_-adrenergic, 5-HT_1_/5-HT_2_, and 5-HT_3_ receptors. Thus, we suggest that co-treatment with BVA and VLX could be used as an alternative novel therapeutic strategy for paclitaxel-induced peripheral neuropathy.

## 5. Materials and Methods

### 5.1. Animals

Six-week-old male C57BL/6j mice (18–20 g at the time of purchase; Daehan Biolink, Chungbuk, Korea) were bred four per colony cage on sawdust bedding with unrestricted access to water and food under controlled room temperature at 23 ± 2 °C. Artificial lighting was maintained on a constant 12-h light-dark cycle (light cycle: 7 AM to 7 PM). Animals were accustomed to the behavioral testing circumstances with handling procedures performed by the same experimenter 1 week before the beginning of the experiments. The animal experimental protocols were approved by the Kyung Hee University Animal Care and Use Committee (KHUASP (SE) 19-011; approved 18 January 2019).

### 5.2. Assessment of Allodynia Behavior

Mice were enclosed beneath an inverted clear plastic cage (12 × 8 × 6 cm) atop a metal mesh floor and allowed to acclimate for 30 min, followed by behavioral assessments. The raters were blinded to the drug administrations.

To measure cold allodynia, brisk reactions of the hind paw in response to acetone stimuli were monitored [16,50]. Ten microliters of acetone (Reagents Chemical Ltd., Gyonggi-do, Korea) was applied to the ventral skin on each side three times 10 min apart, and then the frequencies of brisk shaking and licking of the hind paws were monitored for 30 s.

To quantify mechanical allodynia, rapid withdrawals of the hind paw induced by von Frey filament application were estimated [16,50]. A von Frey hair stimulus (bending force: 0.4 g; Linton Instrumentation, Norfolk, UK) was applied once every 10 s vertically at the mid-area of the plantar skin on each side ten times, until the filament bent. Brisk withdrawal numbers from both sides were monitored, and then calculated as a total percentage response.

### 5.3. Paclitaxel Infusion Protocol

Paclitaxel-induced allodynia was established using four i.p. infusions on alternating days (days 0, 2, 4, and 6) with a final dosage of 8 mg/kg (2 mg/kg/day; Sigma) [2]. An equivalent volume of vehicle (100% ethanol: Cremophor EL: PBS, 1:1:58) was given to control mice using the same infusion method [12].

### 5.4. BVA, Venlafaxine, and Combination Therapy

Bee venom (BV; Jayeonsaeng TJ, Kyeonggi-Do, Korea) was dissolved in PBS (20 μL) [12], and then the different dosages of BV (0.25, 1.0, and 2.5 mg/kg) and vehicle were subcutaneously injected on ST36 (Zusanli acupoint) of the right hind limb located on the anterior tibial muscle area or the neck, one different body area [16].

Venlafaxine (VLX; Sigma, St. Louis, USA) was dissolved in normal saline (NS) at three different concentrations (1, 4, and 6 mg/mL) and was i.p. administered at dosages of 10, 40, or 60 mg/kg, respectively [23]. The vehicle group received the same volume of NS.

In the subsequent combination therapy study, the mice showing allodynic behaviors were administered BVA and VLX simultaneously, each given at an intermediate dosage (i.e., 1.0 mg/kg; s.c. and 40 mg/kg; i.p., respectively).

### 5.5. Spinal Antagonist Administration

To examine the spinal mechanisms of the efficacy of the combination therapy (BVA + VLX), the mice in a neuropathic state were arbitrarily assigned to one of six groups: (i) NS; (ii) dimethyl sulfoxide (DMSO; Sigma, St. Louis, USA); (iii) prazosin (α_1_-AR, 10 μg; Sigma, St. Louis, USA); (iv) idazoxan (α_2_-AR, 10 μg; Sigma, St. Louis, USA); (v) methysergide (5-HT_1_ and 5-HT_2_ receptor, 10 μg; Tocris); or (vi) MDL-72222 (5-HT_3_ receptor, 15 μg; Tocris). The dosages of antagonists were determined according to previously published protocols [23,43,51]. MDL-72222 was dissolved in 20% DMSO [51], and other antagonists were dissolved in NS. Antagonists or vehicles (5 µL) were administered i.t. 20 min prior to combination treatment. Briefly, in a prone position, the allodynic mice were subjected to inhalation anesthesia with isoflurane 2–2.5% in a mixture of oxygen and nitrous oxide (volume 1:1) [23]. Then, a Hamilton syringe needle (10 μL; Hamilton Co., Reno, city, NEV, USA) was inserted vertically into the subarachnoid space (L_5_–L_6_), followed by i.t. administration as previously described [52].

### 5.6. Statistics

Statistical analysis was conducted using Prism 7.0 (GraphPad software, La Jolla, CA, USA). All data are presented as mean ± S.E.M, and *p* < 0.05 was considered significant. Two-way ANOVA followed by Bonferroni’s multiple comparison test was used for statistical analysis.

## References

[1] Hershman D.L., Lacchetti C., Loprinzi C.L. (2014). Prevention and management of chemotherapy-induced peripheral neuropathy in survivors of adult cancers: American Society of Clinical Oncology clinical practice guideline summary. J. Oncol. Pract..

[2] Polomano R.C., Mannes A.J., Clark U.S., Bennett G.J. (2001). A painful peripheral neuropathy in the rat produced by the chemotherapeutic drug, paclitaxel. Pain.

[3] Dougherty P.M., Cata J.P., Cordella J.V., Burton A., Weng H.-R. (2004). Taxol-induced sensory disturbance is characterized by preferential impairment of myelinated fiber function in cancer patients. Pain.

[4] Boyette-Davis J.A., Hou S., Abdi S., Dougherty P.M. (2018). An updated understanding of the mechanisms involved in chemotherapy-induced neuropathy. Pain Manag..

[5] Chaparro L.E., Wiffen P.J., Moore R.A., Gilron I. (2012). Combination pharmacotherapy for the treatment of neuropathic pain in adults. Cochrane Database Syst. Rev..

[6] Eisenberg E., Suzan E. (2014). Drug combinations in the treatment of neuropathic pain. Curr. Pain Headache Rep..

[7] Gilron I., Jensen T.S., Dickenson A.H. (2013). Combination pharmacotherapy for management of chronic pain: From bench to bedside. Lancet Neurol..

[8] Seo B.-K., Han K., Kwon O., Jo D.-J., Lee J.-H. (2017). Efficacy of Bee Venom Acupuncture for Chronic Low Back Pain: A Randomized, Double-Blinded, Sham-Controlled Trial. Toxins.

[9] Zhang S., Liu Y., Ye Y., Wang X.-R., Lin L.-T., Xiao L.-Y., Zhou P., Shi G.-X., Liu C.-Z. (2018). Bee venom therapy: Potential mechanisms and therapeutic applications. Toxicon.

[10] Park J.-W., Jeon J.-H., Yoon J., Jung T.-Y., Kwon K.-R., Cho C.-K., Lee Y.-W., Sagar S., Wong R., Yoo H.-S. (2012). Effects of sweet bee venom pharmacopuncture treatment for chemotherapy-induced peripheral neuropathy: A case series. Integr. Cancer Ther..

[11] Yoon J., Jeon J.-H., Lee Y.-W., Cho C.-K., Kwon K.-R., Shin J.-E., Sagar S., Wong R., Yoo H.-S. (2012). Sweet bee venom pharmacopuncture for chemotherapy-induced peripheral neuropathy. J. Acupunct. Meridian Stud..

[12] Choi J., Jeon C., Lee J.H., Jang J.U., Quan F.S., Lee K., Kim W., Kim S.K. (2017). Suppressive Effects of Bee Venom Acupuncture on Paclitaxel-Induced Neuropathic Pain in Rats: Mediation by Spinal α2-Adrenergic Receptor. Toxins.

[13] Choi S., Chae H.K., Heo H., Hahm D.-H., Kim W., Kim S.K. (2019). Analgesic Effect of Melittin on Oxaliplatin-Induced Peripheral Neuropathy in Rats. Toxins.

[14] Yeo J.-H., Yoon S.-Y., Kwon S.-K., Kim S.-J., Lee J.-H., Beitz A.J., Roh D.-H. (2016). Repetitive acupuncture point treatment with diluted bee venom relieves mechanical allodynia and restores intraepidermal nerve fiber loss in oxaliplatin-induced neuropathic mice. J. Pain.

[15] Archer T., Jonsson G., Minor B.G., Post C. (1986). Noradrenergic-serotonergic interactions and nociception in the rat. Eur. J. Pharmacol..

[16] Kim W., Kim M.J., Go D., Min B.-I., Na H.S., Kim S.K. (2016). Combined effects of bee venom acupuncture and morphine on oxaliplatin-induced neuropathic pain in mice. Toxins.

[17] Zeng Y.-J., Lin Y.-H., Wang Y.-C., Chang J.-H., Wu J.-H., Hsu S.-F., Tsai S.-Y., Lin C.-H., Wen Y.-R. (2018). Laser acupuncture-induced analgesic effect and molecular alterations in an incision pain model: A comparison with electroacupuncture-induced effects. Lasers Med Sci..

[18] Lu K.-W., Hsu C.-K., Hsieh C.-L., Yang J., Lin Y.-W. (2016). Probing the effects and mechanisms of electroacupuncture at ipsilateral or contralateral ST36–ST37 acupoints on CFA-induced inflammatory pain. Sci. Rep..

[19] Zuo C.-Y., Lv P., Zhang C.-S., Lei R.-X., Zhou W., Wu Q.-F., Luo L., Tang Y., Yin H.-Y., Yu S.-G. (2019). Ipsi-and contralateral moxibustion generate similar analgesic effect on inflammatory pain. Evid. Based Complementary Altern. Med..

[20] Finnerup N.B., Attal N., Haroutounian S., McNicol E., Baron R., Dworkin R.H., Gilron I., Haanpää M., Hansson P., Jensen T.S. (2015). Pharmacotherapy for neuropathic pain in adults: A systematic review and meta-analysis. Lancet Neurol..

[21] Deng Y., Luo L., Hu Y., Fang K., Liu J. (2015). Clinical practice guidelines for the management of neuropathic pain: A systematic review. BMC Anesthesiol..

[22] Trouvin A.-P., Perrot S., Lloret-Linares C. (2017). Efficacy of venlafaxine in neuropathic pain: A narrative review of optimized treatment. Clin. Ther..

[23] Li D., Lee J.H., Choi C.W., Kim J., Kim S.K., Kim W. (2019). The Analgesic Effect of Venlafaxine and Its Mechanism on Oxaliplatin-Induced Neuropathic Pain in Mice. Int. J. Mol. Sci..

[24] Kerckhove N., Collin A., Condé S., Chaleteix C., Pezet D., Balayssac D. (2017). Long-term effects, pathophysiological mechanisms, and risk factors of chemotherapy-induced peripheral neuropathies: A comprehensive literature review. Front. Pharmacol..

[25] Starobova H., Vetter I. (2017). Pathophysiology of chemotherapy-induced peripheral neuropathy. Front. Mol. Neurosci..

[26] Mao J., Gold M.S. (2011). Combination drug therapy for chronic pain: A call for more clinical studies. J. Pain.

[27] Cherniack E.P., Govorushko S. (2018). To be or not to be: The potential efficacy and safety of bee venom acupuncture in humans. Toxicon.

[28] Chen J., Lariviere W.R. (2010). The nociceptive and anti-nociceptive effects of bee venom injection and therapy: A double-edged sword. Prog. Neurobiol..

[29] Moulin D., Boulanger A., Clark A., Clarke H., Dao T., Finley G., Furlan A., Gilron I., Gordon A., Morley-Forster P. (2014). Pharmacological management of chronic neuropathic pain: Revised consensus statement from the Canadian Pain Society. Pain Res. Manag..

[30] Kus T., Aktas G., Alpak G., Kalender M.E., Sevinc A., Kul S., Temizer M., Camci C. (2016). Efficacy of venlafaxine for the relief of taxane and oxaliplatin-induced acute neurotoxicity: A single-center retrospective case–control study. Support. Care Cancer.

[31] Durand J.-P., Guillevin L., Goldwasser F. (2005). Clinical activity of venlafaxine and topiramate against oxaliplatin-induced disabling permanent neuropathy. Anti-Cancer Drugs.

[32] Cegielska-Perun K., Bujalska-Zadrożny M., Tatarkiewicz J., Gąsińska E., Makulska-Nowak H.E. (2013). Venlafaxine and neuropathic pain. Pharmacology.

[33] Pedersen L.H., Nielsen A.N., Blackburn-Munro G. (2005). Anti-nociception is selectively enhanced by parallel inhibition of multiple subtypes of monoamine transporters in rat models of persistent and neuropathic pain. Psychopharmacology.

[34] Folkesson A., Honoré P.H., Bjerrum O.J. (2010). Co-administered gabapentin and venlafaxine in nerve injured rats: Effect on mechanical hypersensitivity, motor function and pharmacokinetics. Scand. J. Pain.

[35] Lim B.-S., Moon H.J., Li D.X., Gil M., Min J.K., Lee G., Bae H., Kim S.K., Min B.-I. (2013). Effect of bee venom acupuncture on oxaliplatin-induced cold allodynia in rats. Evid.-Based Complementary Altern. Med..

[36] Hache G., Guiard B., Nguyen T., Quesseveur G., Gardier A., Peters D., Munro G., Coudoré F. (2015). Antinociceptive activity of the new triple reuptake inhibitor NS 18283 in a mouse model of chemotherapy—Induced neuropathic pain. Eur. J. Pain.

[37] Hanna M., O’Brien C., Wilson M.C. (2008). Prolonged-release oxycodone enhances the effects of existing gabapentin therapy in painful diabetic neuropathy patients. Eur. J. Pain.

[38] Gilron I., Bailey J.M., Tu D., Holden R.R., Weaver D.F., Houlden R.L. (2005). Morphine, gabapentin, or their combination for neuropathic pain. New Engl. J. Med..

[39] Hajhashemi V., Banafshe H.R., Minaiyan M., Mesdaghinia A., Abed A. (2014). Antinociceptive effects of venlafaxine in a rat model of peripheral neuropathy: Role of alpha2-adrenergic receptors. Eur. J. Pharmacol..

[40] Millan M.J. (2002). Descending control of pain. Prog. Neurobiol..

[41] Yoshimura M., Furue H. (2006). Mechanisms for the anti-nociceptive actions of the descending noradrenergic and serotonergic systems in the spinal cord. J. Pharmacol. Sci..

[42] Liu Z.-Y., Zhuang D.-B., Lunderberg T., Yu L.-C. (2002). Involvement of 5-hydroxytryptamine1A receptors in the descending anti-nociceptive pathway from periaqueductal gray to the spinal dorsal horn in intact rats, rats with nerve injury and rats with inflammation. Neuroscience.

[43] Godoy M.C.M., Fighera M.R., Souza F.R., Flores A.E., Rubin M.A., Oliveira M.R., Zanatta N., Martins M.A., Bonacorso H.G., Mello C.F. (2004). α2-Adrenoceptors and 5-HT receptors mediate the antinociceptive effect of new pyrazolines, but not of dipyrone. Eur. J. Pharmacol..

[44] Kawamura M., Ohara H., Go K., Koga Y., Ienaga K. (1998). Neurotropin induces antinociceptive effect by enhancing descending pain inhibitory systems involving 5-HT3 and noradrenergic α2 receptors in spinal dorsal horn. Life Sci..

[45] Lockwood S.M., Bannister K., Dickenson A.H. (2019). An investigation into the noradrenergic and serotonergic contributions of diffuse noxious inhibitory controls in a monoiodoacetate model of osteoarthritis. J. Neurophysiol..

[46] Nakagawa I., Omote K., Kitahata L.M., Collins J., Murata K. (1990). Serotonergic mediation of spinal analgesia and its interaction with noradrenergic systems. Anesthesiology.

[47] Yoshimura M., Furue H. (2017). In Vivo electrophysiological analysis of mechanisms of monoaminergic pain inhibitory systems. Pain.

[48] Wiesenfeld-Hallin Z. (2005). Sex differences in pain perception. Gend. Med..

[49] Beery A.K. (2018). Inclusion of females does not increase variability in rodent research studies. Curr. Opin. Behav. Sci..

[50] Li D., Lee Y., Kim W., Lee K., Bae H., Kim S.K. (2015). Analgesic effects of bee venom derived phospholipase A2 in a mouse model of oxaliplatin-induced neuropathic pain. Toxins.

[51] Lee J.-H., Li D.X., Yoon H., Go D., Quan F.S., Min B.-I., Kim S.K. (2014). Serotonergic mechanism of the relieving effect of bee venom acupuncture on oxaliplatin-induced neuropathic cold allodynia in rats. BMC Complement. Altern. Med..

[52] Hylden J.L., Wilcox G.L. (1980). Intrathecal morphine in mice: A new technique. Eur. J. Pharm..

